# The Need to Widen the Concept of Health and to Include the Spiritual Dimension

**DOI:** 10.3389/ijph.2024.1606648

**Published:** 2024-04-04

**Authors:** Ursula Wüthrich-Grossenbacher

**Affiliations:** Centre for African Studies, Faculty of Humanities and Social Sciences, University of Basel, Basel, Switzerland

**Keywords:** definition of health, traditional medicine, WHO, religion and spirituality, collaborative approach

## Abstract

For many, the World Health Organization’s (WHO) definition of health does not reflect their own understanding of health, because it lacks aspects such as spiritual wellbeing. Responding to these concerns, the WHO called in 2023 for a vision of health that integrates physical, mental, psychological, emotional, spiritual, and social wellbeing. To date, medical practitioners are often reluctant to consider spiritual aspects, because of a perceived lack of statistical evidence about the strength of relations. Research on this topic is emerging. A recent study among 800 young people living with HIV in Zimbabwe showed how study participants navigated three parallel, at times contradicting health systems (religious, traditional, medical). Conflicting approaches led to multifaceted dilemmas (= spiritual struggles), which were significantly related to poorer mental and physical health. This illustrates the need for inclusion of spiritual aspects for health and wellbeing in research, and of increased collaboration between all stakeholders in healthcare.

## Introduction

For many years the World Health Organization (WHO) has grappled with its definition of health, recognizing both its utopian perspective and its limitations. Repeatedly, WHO member states have called for a more realistic and holistic definition, and in particular for the inclusion of the spiritual dimension. One such attempt was made in 1984, when the 37th World Health Assembly adopted a resolution calling for the inclusion of the spiritual dimension in the Global Strategy for Health for All by the year 2000. Although the discussions in the World Health Assembly showed an appreciation for a spiritual dimension of health, they also revealed some difficulties, such as the question of whether a constitutional amendment was the right tool, and the ambiguity of the term “spiritual” [1]. Hence, the constitutional amendment had to be postponed. Indeed, there are many different definitions for the two terms “religion” and “spirituality” and as Vader rightly points out, although there will be “some overlap between the definition of spiritual health and measurements of “spirituality, religiousness, and personal beliefs” that have been proposed, we cannot assume that they are synonymous” [2]. In this paper the term “religion” refers to organised and/or shared faith practice or belief and the term “spirituality” refers to the way people relate to the transcendent, including traditional practices.

The WHO quality of life measure (WHOQOL) was another attempt by the WHO to widen the horizon for different ontologies of health and wellbeing. It was developed by fifteen international field centres in an attempt to develop a quality of life assessment that would be applicable cross-culturally [3]. The discussions involved input from stakeholder of different faith traditions and resulted in a nuanced and widely shared consensus of what really matters in life [1]. The WHOQOL has been used widely in many different settings and conditions (often in its abbreviated version) [4, 5]. However, its cross-cultural suitability remains a challenge [1].

In a continuing effort to recognize the spiritual dimension of health, the WHO is increasingly using the term “spiritual wellbeing.” In the 2005 Bangkok Charter for Health Promotion in a Globalized World, the WHO states that health is a fundamental right and that health promotion is based on this, offering a positive and inclusive concept of health that includes mental and spiritual wellbeing [6]. Despite these efforts, the call for a new definition of health continues. In 2016 leaders or representatives of indigenous peoples, anthropologists and physicians from many cultural backgrounds (Amazonia, Patagonia, Papua New Guinea, Inuit, North-American Indian, Sub-Saharan Africa, India, China, Melanesia and Polynesia) wrote an open letter to the WHO calling for a new definition of health. They argued that the WHO definition of health was outdated, utopian rather than pragmatic, and inappropriate for a large part of the world’s population. They proposed several key concepts that the WHO should reintegrate into a new definition of health: human equilibrium in nature, accepted spirituality, and adaptation [7].

The WHO responded to these concerns by calling for a framework for achieving wellbeing in 2022. A draft of this framework was presented to the WHO Executive Board in 2023. It incorporates many of the demands of the above letter. It explicitly states that the concept of ‘wellbeing societies’ partly stems from awareness and appreciation of indigenous knowledge systems, and that “wellbeing societies” should apply policies and approaches that are underpinned by, among others: a positive vision of health that integrates physical, mental, psychological, emotional, spiritual and social wellbeing; the principles of human rights, social and environmental justice, solidarity, gender and inter-generational equity, and peace; and new indicators of success, beyond gross domestic product, that take account of individual and societal wellbeing and lead to new priorities for public spending on health [8].

This promising approach now has to be implemented. As with the WHOQOL, other WHO initiatives, like the WHO Traditional Medicine Strategy 2014–2023, are equally well grounded theoretically but slow in their implementation. Its need is illustrated in a recent study among 800 young people living with HIV in Zimbabwe. Young people living with HIV in Zimbabwe consulted traditional practitioners and medical clinics in parallel. A lack of cooperation between the stakeholders of the two systems led to potentially conflicting approaches [9].

## Lack of Holistic Approaches Respecting Local Ontologies of Health and WellBeing: Health Implications

`The findings of the above-mentioned study illustrate the consequences of the continued hesitancy of medical HIV care providers to integrate cultural and religious/spiritual (R/S) aspects into their programmes. In Zimbabwe, many young people have a dual belief system. While officially associated with Christianity or any other religion, traditional beliefs and practices remain important. In this context, wellbeing derives from the cultural understanding of the role of family, community, and the spiritual world in human welfare. Illnesses, and especially chronic conditions like an HIV infection, may be understood as having physical, mental, social, spiritual, and supernatural causes. Thus, the right treatment depends on the perceived cause of an illness. Healing extends beyond physical symptoms to address social and spiritual aspects as well [10]. Yet, public health initiatives for HIV care in Zimbabwe largely ignore local ontologies of health and wellbeing. This left the study participants alone and at times overwhelmed in a complex situation with three different health systems (traditional practitioners, religious healers, biomedical care). Young people living with HIV in Zimbabwe navigated three, sometimes incompatible health systems, and faced conflicting views and approaches. This had a significant impact on their risk taking, health seeking behaviour, and HIV health outcomes [11].

Furthermore, by focusing solely on biomedical care, the important contribution of traditional practitioners, religious healers, and communities to public healthcare was not only ignored but challenged. In our study, participants affirmed better accessibility, affordability, and cultural/spiritual relevancy of traditional practitioners and religious healers. Yet, the contributions of traditional practitioners and religious healers were usually not recognised in HIV care. They were largely excluded from participation in economic, academic, and government entities and health policy making. Traditional practitioners and religious healers and communities complemented, but at times also compromised the biomedical therapy. This caused multifaced dilemmas for the young people in this study. Nearly fifty percent of study participants reported religious/spiritual struggles [12]. The four dimensions of religious/spiritual struggles experienced by study participants were religious alienation, religious confusion, religious doubts or conflicts, and high religious zeal that led to frustration. These religious/spiritual struggles were significantly related to higher HIV load, higher risk of mental health issues, and higher prevalence of opportunistic infections [13].

The relevance of religious and spiritual struggles was also found in so called secularized societies, like Czechoslovakia [14] and Switzerland [15]. The study in Switzerland involved 1,359 German-speaking participants, primarily university students. Although the prevalence of religious/spiritual struggles was relatively low, researchers found strong associations between religious/spiritual struggles and depression [15].

## Reasons for the Reluctance of Integrating R/S Aspects Into HealthCare Practice

One main argument against the inclusion of R/S issues in healthcare, is the perceived lack of possible metrics to measure spirituality or spiritual wellbeing. In my own research, following argument was the most common in conversations with medical doctors: “Either spirituality should be defined and measured in traditional terms as a unique, uncontaminated construct, or it should be eliminated from use in academic research” [16]. Furthermore, medical doctors tend to dismiss the increasing evidence of the important influence of R/S on therapeutic itineraries and health outcomes because most of these studies are qualitative in nature or are based on descriptive statistics, which is not considered strong evidence. This is exemplified by the United Nations Children’s Fund’s (UNICEF) conclusion to its own analysis of Zimbabweans’ Multiple Indicator Cluster Survey (MICS) in 2014. On the one hand, UNICEF stated that “the analysis of MICS 2014 data on religion offers evidence on its influence on health, educational and social outcomes,” and on the other hand, UNICEF still called for future studies to “apply advanced statistical analysis including multi-level logistic regression to test and reveal the strengths of different causes” [17]. This ambiguity is a good example of a global phenomenon. Despite apparent evidence of the importance of alternative ontologies of health and R/S issues, scientists, and especially medical doctors, adhere to concepts and categories that are rooted in rationalism.

## Possible Benefits of Integrating R/S Aspects Into Public Health Approaches

In the context of Zimbabwe, integrating local ontologies of health and wellbeing would automatically necessitate the collaboration of the three parallel health systems. While there are existing challenges and seemingly incongruent beliefs and practices, they may not be unsurmountable. What is needed, is a dialogue at eye level between the different stakeholders. A collaboration of the different stakeholders has the potential to mitigate compromising and contradicting healthcare approaches and to increase areas of complementation. In fact, the working together of all three health sectors could achieve, what none of them could achieve alone, namely constitute a wellbeing society with a positive vision of health that integrates physical, mental, psychological, emotional, spiritual and social wellbeing [8].

I argue that above findings are relevant beyond the specific context of the Zimbabwean study:

Firstly, because ontologies of health that include R/S aspects are not non-Western per-se, but present and documented worldwide. Souček illustrated this in a recent study that compared R/S healing activities in Slovakia and India during the COVID-19 pandemic [18]. While resorting to R/S in times of crisis was expected in India, it was also evident in Slovakia. During the pandemic, religious healing rituals like prayer, speaking in tongues, fasting, or the veneration of corporal relics of saints became very important in Slovakia. Souček concludes that “People living in so-called modern secular societies are, to a substantial extent, inclined to use certain forms of religious and ritualistic practices when facing unprecedented difficulties and challenges. Certain archaic practices thought to have completely vanished from our modern societies seem to be present in the everyday lives of supposedly “modern” people” [18].

Secondly, ontologies of health that include R/S aspects are not “unscientific” per-se. Studies, like the Zimbabwean study mentioned above [13], demonstrate that R/S aspects, including traditional practices and beliefs, can be defined, measured, quantified, and empirically analysed to find the strengths of different causes. Very importantly, the study was able to show the significant relation between R/S aspects and current blood results.

Thirdly, initiatives to incorporate spiritual and religious systems of belief and practice, especially in psychiatric practice, are promising. The findings of a multi-level meta-analysis that compared randomized controlled studies of the efficacy between R/S-based and regular treatments in mental healthcare settings suggest that “treatments with a focus on religious and spiritual issues are more efficacious than non-R/S-based therapy” [19]. Another study in a more secularized context in 2016 examined the impact of pastoral care interventions on the mental and emotional health of inpatients at a private psychiatric hospital in Sidney. A vast majority of patients with a length of stay of 1–4 weeks reported benefits during and after their meeting (s) with a pastoral care practitioner, regardless of their religious beliefs. Some of these patients only had one meeting of 30 min. Patients reported that they were able to talk about things that they would not be comfortable discussing with other health professionals. This was significantly associated with lowered anxiety levels [20]. Evidently, more research on this topic is required as these study results need to be further validated and expanded. But they hint at a possibility of a, at the same time, more holistic, less time consuming, resource saving, and effective care approach.

## Conclusion

The continued hesitancy of many medical practitioners and public health policymakers to integrate R/S into the public health approach illustrate, that new negotiations of the concepts of health and healthcare are not merely the task of the WHO, but of—among others—those with lived experience, all relevant health practitioners, and scientists from all walks of life and cultural, religious, and local contexts. For the future recognition and integration of patients’ R/S resources (including traditional medical practice and religious healers) and R/S needs, a change of attitude is necessary. Instead of copying the approaches, methods, categories, concepts, and presumptions from the past, scientists and health practitioners worldwide need to engage with local environments and ontologies and develop new methods and concepts of health and healthcare. Such new concepts should originate in dialogues, collaborations, exchange, and participation from, with, and among people of all cultural, religious, and educational backgrounds. Only then, will it be possible to mitigate negative impacts of contradicting ontologies of health, and possibly improve public healthcare to become culturally more relevant, religiously more sensitive, more effective, and possibly also save resources, and improve access to healthcare for all.
